# Correction to “Maternal neural responses to infant cries and faces: relationships with substance use”

**DOI:** 10.3389/fpsyt.2012.00115

**Published:** 2013-02-07

**Authors:** Nicole Landi, Jessica Montoya, Hedy Kober, Helena J. V. Rutherford, W. Einar Mencl, Patrick D. Worhunsky, Marc N. Potenza, Linda C. Mayes

**Affiliations:** ^1^Child Study Center, Yale UniversityNew Haven, CT, USA; ^2^Department of Psychiatry, Yale UniversityNew Haven, CT, USA; ^3^Haskins LaboratoriesNew Haven, CT, USA

The article “Maternal neural responses to infant cries and faces: relationships with substance use” by Landi et al. (2011), 2, 1–13 contains an error. Specifically Figures 1 and 2, were partially swapped, with Figure 1 displaying images in response to cries, and Figure 2 displaying responses to faces. Both figures are presented correctly here, with the appropriate captions. All of the text and the tables in the manuscript are correct, this was the only error. We regret this mistake.

**Figure 1:**
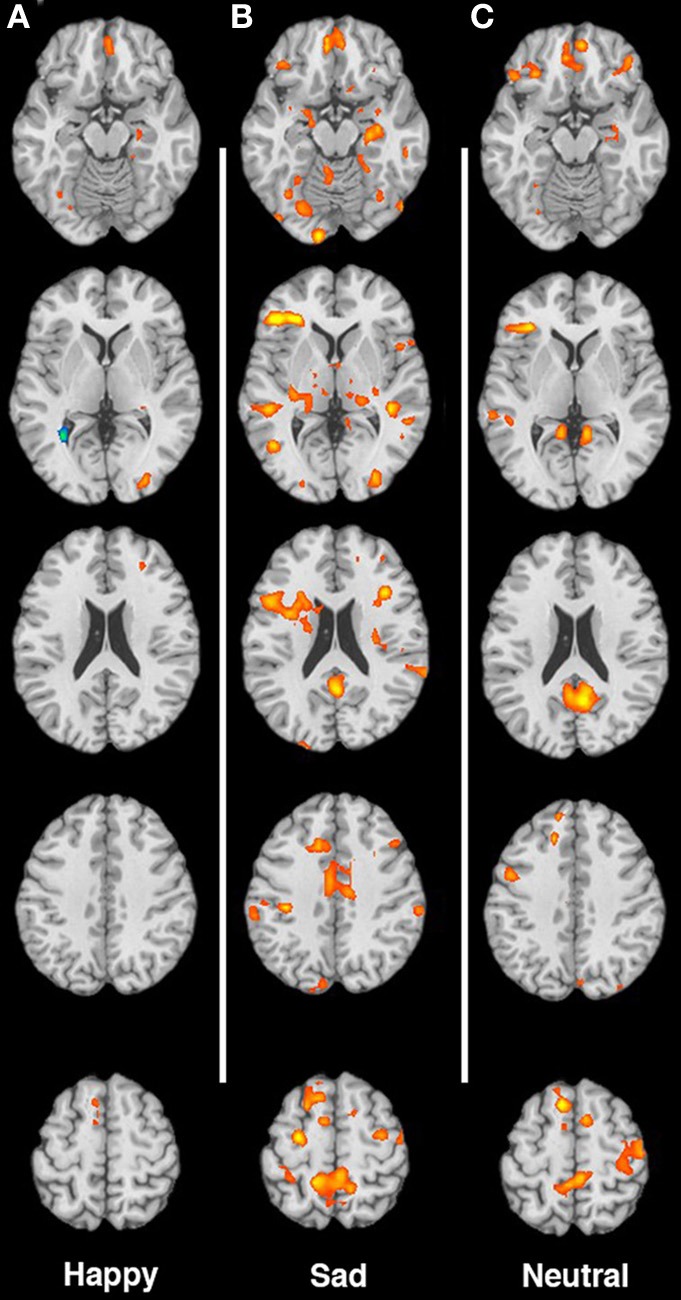
**(A–C)** Contrast of non-using > substance-using mothers for happy, sad, and neutral faces, respectively. Images are shown in neurological convention, with the left hemisphere on the left faces, respectively. Images are shown in neurological convention, with the left hemisphere on the left side of each image. Slices are at MNI z-coordinate locations −19, −1, +17, +35, and +53 mm.

**Figure 2:**
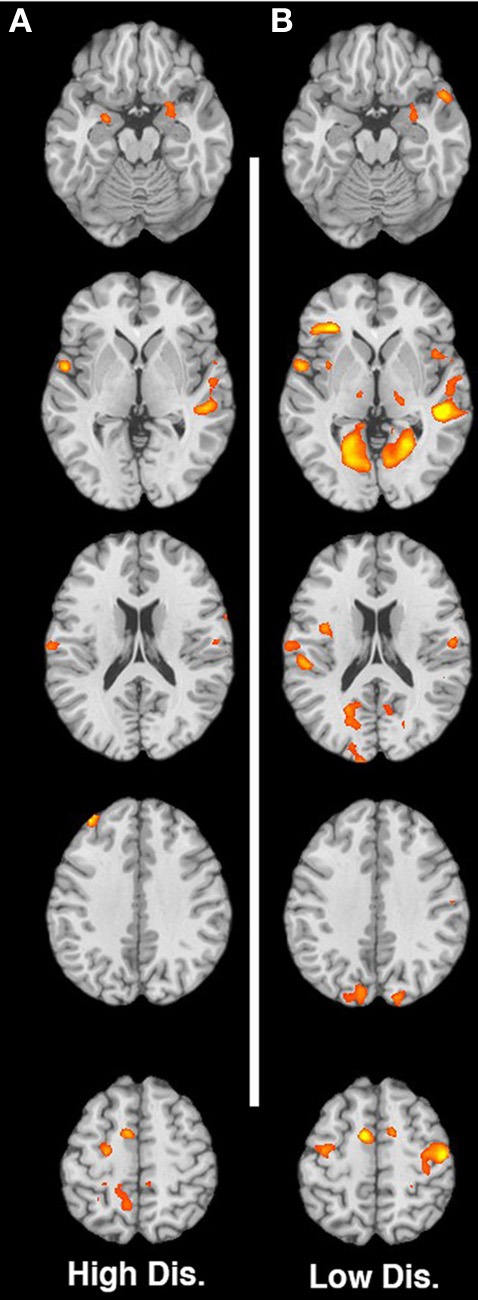
**(A–B)** Contrast of non-using > substance-using for low-distress and high-distress cries respectively. Slices are at MNI z-coordinate locations −16, +2, +20, +38, and +56 mm.

